# Is the bone marrow microenvironment the hidden catalyst in malignant haematopoiesis?

**DOI:** 10.1038/s41375-025-02630-6

**Published:** 2025-04-29

**Authors:** Syed A. Mian, Steven Ngo, Dominique Bonnet

**Affiliations:** Haematopoietic Stem Cell Lab, The Francis Crick Institute, London, UK

**Keywords:** Cancer microenvironment, Haematopoietic stem cells

**Fig. 1:**
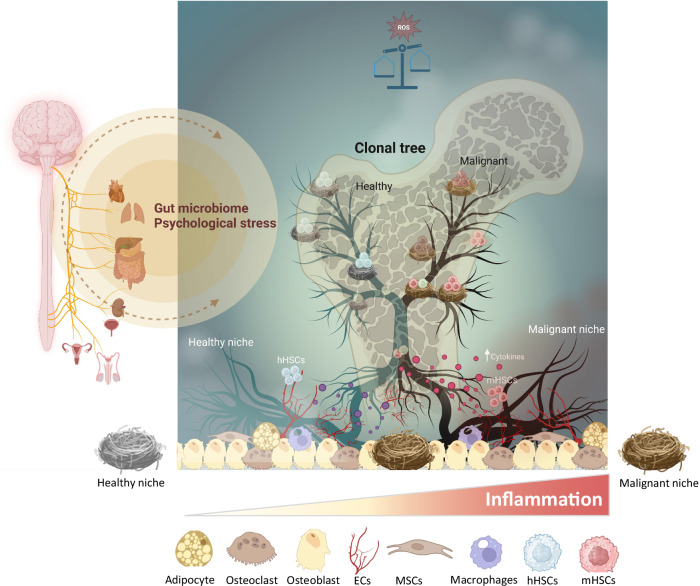
The bone marrow microenvironment plays an instructive role in shaping clonal architecture. During the early pre-disease stages, microenvironmental cells can influence haematopoietic stem cells—both normal and malignant—through direct cell-cell contact and cytokine secretion. These signals help regulate cellular responses, potentially driving disease progression. Healthy and malignant haematopoietic stem cells may occupy distinct micro-niches or share niche spaces, influencing their behaviour and interactions within the bone marrow. Gut microbiome as well as psychological stress may also influence the regulation of haematopoiesis in the bone marrow. ECs Endothelial cells, hHSC Healthy haematopoietic stem cells, mHSC Malignant haematopoietic stem cells, MSCs Mesenchymal stroma cells. Created with BioRender.com.

Over the past decades, a recurring question in haematologic research has been the role of the bone marrow niche in myeloid malignancies such as Myelodysplastic Syndromes (MDS) and Acute Myeloid Leukaemia (AML). This highly controversial debate has centred on whether—and to what extent—the bone marrow microenvironment (BMME) actively drives the transformation of healthy hematopoietic stem cells (HSCs) into malignant counterparts or simply provides a permissive environment where cancer cells thrive (Fig. 1).

Comprising over 20 distinct cell types, the bone marrow is a complex tissue that is known to tightly control the delicate balance between self-renewal and differentiation, as well as dormancy or proliferation of the HSCs [1, 2]. The clonal heterogeneity of haematological neoplasms, particularly AML and MDS, has been well characterized through the discovery of chromosomal abnormalities and somatic mutations affecting RNA splicing, epigenetic machinery, cell signalling factors and transcription factor genes. These mutations are primarily described in hematopoietic cells and some have been traced back to phenotypically defined HSCs. It is now recognized that the bone marrow in these malignancies inhabits multiple genetically distinct subclones, often following a branching, multi-clonal, or ancestral evolutionary trajectory, leading to significant intra-tumour heterogeneity over the course of the disease [3–5].

Reports implicating the BMME as a primary driver of disease remain rare, partly due to historically limited knowledge of the specific cell types within this complex tissue. Recent advancements in single-cell technologies have begun to map the ‘bone marrow metropolis’ [6, 7], yet our understanding of its functional composition still remains incomplete. The intricate network of inter- and intracellular regulatory mechanisms within this complex tissue remains an active area of exploration, with many pieces of the jigsaw yet to be placed correctly. Specific components within the bone marrow such as mesenchymal stroma cells (MSCs), are well-recognized as key niche players supporting the HSCs [2]. A study by Raajimakers and colleagues demonstrated that genetic modification of osteolineage cells in murine bone marrow disrupts hematopoietic system integrity. Specifically, the targeted deletion of the miRNA-processing enzyme Dicer1 in osteoprogenitor cells—but not in mature osteoblasts—impaired HSC survival, proliferation, and differentiation. This murine model exhibited hematopoietic abnormalities resembling human MDS and ultimately showed leukaemia progression, characterized by distinctive secondary genetic abnormalities in haematopoietic cellular system [8]. Further experimental evidence comes from another murine study reporting that a mutated form of protein tyrosine phosphatase SHP2 (encoded by PTPN11), a positive regulator of the RAS signalling pathway, in MSCs and osteoprogenitors, drives the development and progression of myeloproliferative neoplasms (MPN) through severe detrimental effects particularly on HSCs. Notably, Ptpn11 mutations in BMME cells trigger excessive production of the CC chemokine CCL3, leading to the recruitment of monocytes to the HSC niches. As a result, these HSCs become hyperactivated by elevated levels of interleukin-1β and potentially other proinflammatory cytokines produced by monocytes, ultimately exacerbating MPN phenotype [9]. Similarly, mice deficient for retinoic acid receptor γ (RARγ) in the BMME have been shown to develop MPN-like disease with older mice showing a more pronounced disease phenotype [10]. Increased β-catenin signalling and nuclear accumulation have been reported in MDS/AML patient osteoblasts and these patients showed increased Notch signalling in haematopoietic cells [11]. While these reports describing the role of the BMME are still limited, they provide convincing data supporting the concept of a niche-driven model of oncogenesis. In this multi-step process, the very initial event occurs in the stromal microenvironment, leading to secondary genetic or epigenetic alterations in hematopoietic cells. This perspective is particularly compelling as it challenges the conventional view that cancer initiation is entirely or primarily driven by autonomous mutations within malignant HSCs.

Clinically, donor cell leukaemia (DCL), though rare, serves as an excellent example of niche-driven disease, where leukaemia originates from engrafted donor cells following allogeneic stem cell transplantation (HSCT) [12]. While such cases are infrequent, they also challenge the notion that myeloid cancer initiation and progression are entirely hematopoietic cell-autonomous processes. It is important to note that detailed genomic screening has not been systematically performed to determine whether donor HSCs themselves carry any pre-existing gene mutations. Therefore, studies are needed to investigate if donor cells themselves may have been part of an asymptomatic phase within the donor, characterized by clonal expansion of HSCs—a phenomenon recently termed ‘clonal haematopoiesis of indeterminate potential’ (CHIP) [13, 14]. Even so, in such circumstances, it is conceivable that pre-existing donor CHIP mutations may only manifest into DCL in response to the highly inflammatory conducive niche present in the host.

Interestingly, the risk of progression from CHIP to MDS to AML is significantly heterogeneous among the patients despite the fact that the precursor states of MDS and AML are associated with similar gene mutations conferring a clonal advantage [15, 16]. This raises the question of whether donor cells have an intrinsic genomic predisposition that makes them more susceptible to acquiring additional abnormalities in response to the recipient’s ‘mutation-promoting’ BMME. Disease progression may thus depend on niche cells that either permit or inhibit further clonal expansion. The phenomenon of DCL clearly raises the possibility that the BMME itself may contribute to leukemogenesis, either by inducing genetic or epigenetic alterations in previously healthy donor cells or by creating a permissive and selective pressure that favours malignant clonal selection. These cases underscore the potential role of extrinsic factors within the BMME in shaping disease evolution. For instance, what role do elevated levels of reactive oxygen species (ROS) production play? How do exosomes mediate both near- and long-distance intercellular communication? And how does direct cell-cell contact influence the priming of the BMME for leukemogenesis? These questions highlight the need for a deeper understanding of how the human BMME interacts with hematopoietic cells in both normal and malignant states.

In the real world, one must ask: What part, if any, does stress—induced by either HSCT conditioning, repeated viral infections, blood loss or inflammaging—play within the complex BMME in activating clonal hematopoietic stem cells, whether it will be individual’s own HSCs or transplanted donor HSCs? It is well established that, in murine models, inflammatory signalling mediated by cytokines such as interferon-α, tumour necrosis factor-α can activate HSCs, including those in a dormant state [17, 18]. However, it remains unknown whether these cytokines are produced by the infected hematopoietic cell populations, whether niche cells respond to stress by producing them, or if both mechanisms operate in some sort of a feedback loop. In the context of HSCT, a damaged BMME following intensive conditioning regimens may induce a stress response, particularly with overproduction of ROS and inflammatory markers that are detectable for decades later, as has been reported in cases of atomic bomb survivors and Chernobyl workers [19–21]. It is crucial to determine whether these continuous chronic inflammatory insults induce persistent changes in the epigenetic memory of the BMME, ultimately impairing stem cell function and accelerating age-related decline in tissue regeneration.

Likewise, therapy-induced remodelling of the BMME at a cellular level may lead to abnormal activation of pre-existing clonal haematopoietic cells. Recently, it has becoming increasingly apparent that the bone marrow vasculature is a heterogenous and highly specialised component of the bone marrow niche [22, 23]. Similar to the MSC, the vasculature also plays a critical role in regulating haematopoiesis and HSC maintenance, either through providing key regulatory signals (such as Notch ligands) or regulating local oxygen concentrations to promote HSC proliferation or quiescence [24–26]. The endothelial cell compartment has shown to be particularly sensitive to therapeutic agents commonly used to treat MDS and AML, such as myeloablative agents or chemotherapy [27, 28]. Consequently, the tightly controlled regulation of localized oxygen levels as well as production of niche factors from the endothelial cells can be significantly compromised and potentially lead to abnormal HSC activation and selection of HSC clonal populations.

Beyond the effectiveness of chemotherapy in treating haematological neoplasms, there is emerging challenge related to increasing incidence of therapy-related myeloid neoplasms (t-MN) in long-term solid cancer survivors [29]. The prevailing hypothesis suggests these t- MNs are HSC autonomous disorders, where cytotoxic therapies induce genome-wide DNA damage in HSCs that drive the initiation and progression to t-MN. However, it is important to note that only a subset of solid cancer patients treated with chemotherapy or radiation develop t-MN, indicating the influence of other factors such as genetic predisposition, BMME, aging or pre-existing CHIP clones in t-MN pathogenesis. Emerging evidence suggests that cytotoxic therapies induce long-term damage in BM MSCs, leading to cellular senescence. This in turn creates a highly proinflammatory microenvironment driven by paracrine and endocrine signalling through senescence-associated secretory phenotype (SASP), as has been reported in t-MN murine model [30] and primary patients [31]. The persistence of senescent cells can lead to a highly proinflammatory microenvironment conducive to the progression of mutated clones and facilitating disease progression to t-MN [31].

Although the outcome of HSCT is largely dictated by genetic factors, most notably human leucocyte antigen (HLA) matching being the critical clinical determinant. However, one must not forget the rest of the genome [32, 33]. It is well established that the genetic polymorphisms, particularly single-nucleotide polymorphisms (SNPs) that represent natural variations within the population, can have a bearing on the function of a gene. Additionally, variations in regulatory elements, such as microRNAs and enhancers within the genome, play a crucial role in modulating immune responses. Consequently, differential expression patterns of messenger RNAs (mRNAs), microRNAs, and SNPs in key genes involved in inflammatory signalling, stress response, and metabolic pathways in the recipient’s BMME cells can profoundly impact donor hematopoietic stem cell reconstitution dynamics. Furthermore, several growth factors, their receptors, and effector molecules function as proto-oncogenes or tumour suppressors [34]. A recipient whose BMME cells harbours a ‘high risk’ (for example, inflammation) SNP profile in these genes can potentially contribute to further driving the selection and expansion of donors malignant HSC clones. Therefore, the reconstituted bone marrow serves as a dynamic ‘battleground’ for cell-cell interactions, where donor and recipient cells engage in a complex interplay at various levels of regulation, each striving to establish dominance over the other. More human studies are required to comprehensively explore these aspects and gain a complete understanding of HSCT reconstitution.

Haematopoietic cells themselves have been shown to be critical in maintaining the bone marrow homoeostasis [2]. To better understand the dynamics of the BMME, a key question emerges: Do malignant HSCs actively recruit and manipulate their microenvironment to support their survival and expansion during the early stages of disease? Specifically, do malignant HSCs directly influence the production or recruitment of niche-supporting cells, such as macrophages, that they recognize as essential for their propagation? Studies have shown that depletion of macrophages in murine models leads to reduction of HSC-supporting cytokines at the bone marrow endosteum and mobilization of HSCs into the peripheral blood and a decline in HSC numbers [35, 36]. Since macrophages are downstream progeny of HSCs, this raises the possibility that malignant HSCs may have the capacity to shape their own niche by generating or modifying key stromal and immune components. Such a dynamic, reciprocal relationship between leukaemic cells and their microenvironment could allow cancer cells to establish a self-sustaining niche that not only protects them from immune surveillance, as well as clearance and therapeutic interventions but also accelerates disease progression.

In conclusion, the growing body of evidence challenges the traditional view that haematological neoplasms are driven solely by intrinsic mutations in HSCs. The complex interactions between malignant HSCs and their BMME, including stromal and immune components, create a dynamic, reciprocal relationship that may not only support the survival and expansion of cancer cells but also influence disease trajectory. The rare but insightful phenomenon of DCL underscores the potential of the microenvironment to contribute to leukemogenesis, either by inducing genetic or complex epigenetic alterations in donor cells or fostering a selective pressure that promotes malignant selection. Another key area of exploration is the role of the gut microbiome and psychological stress in regulating haematopoiesis under homoeostatic and malignant conditions, as well as during reconstitution following HSCT. This regulation may involve mechanisms such as cytokine release mediating immune cell recruitment through feedback loops, and the initiation of emergency haematopoiesis. The concept of maternal foetal microchimerism, a term related to the presence of bidirectional trafficking of cells, cytokines and other biological factors between the mother and foetus, extends to the BMME in intriguing ways. Despite its potential significance, the role of maternal-foetal microchimerism within the BMME remains an underexplored area of research. It is essential to highlight that the majority of the existing literature on BMME has focused on murine models, urgently underscoring the need for more human studies. These should include mutational and epigenetic profiling of patient BMME, investigating BMME characteristics in DCL cases and also direct analysis of bone marrow trephines to examine the role of malignant cell recruitment in both shaping the disease-initiating as well as sustaining disease-propagating niches. As we continue to unravel the intricate network of cellular and molecular players in the BMME, it becomes increasingly evident that targeting this microenvironment may offer a promising therapeutic approach. By better understanding the complex interplay between hematopoietic cells and their surroundings, we may uncover novel strategies to combat haematologic cancers and improve patient outcomes.
